# Selective versus routine lymphadenectomy in the treatment of liver metastasis from colorectal cancer: a retrospective cohort study

**DOI:** 10.1186/s12893-017-0233-y

**Published:** 2017-04-04

**Authors:** Daniel Pindak, Jana Pavlendova, Miroslav Tomas, Jozef Dolnik, Robert Duchon, Juraj Pechan

**Affiliations:** 1 Department of Surgical Oncology, Slovak Medical University, Bratislava, Slovakia; 2 National Cancer Institute, Klenova 1, 833 10 Bratislava 3, Slovakia

**Keywords:** Colorectal, Liver, Metastasis, Lymphadenectomy, Survival

## Abstract

**Background:**

Limited data are available on the importance of routine lymphadenectomy of the hepatoduodenal ligament in the treatment of liver metastasis from colorectal cancer in the literature.

**Methods:**

A single center retrospective cohort study was conducted to evaluate morbidity and long-term survival in patients who had undergone selective versus routine lymphadenectomy during surgery for colorectal liver metastasis. From January 2006 to December 2009, eighty-one patients undergoing radical resection due to liver metastasis from colorectal cancer were included. The combination of two surgical teams with different approaches to hepatoduodenal ligament lymphadenectomy at our institution allowed us to select two cohorts of patients undergoing selective or routine lymphadenectomy.

**Results:**

No significant differences between the cohorts were found in age, American Society of Anesthesiology score or Fong’s prognostic criteria. Patients with pN+ disease had significantly inferior survival compared to patients with pN0 disease (hazard ratio [HR] = 6.33, 95% CI 2.16–18.57, *p* = 0.0001). No significant difference in postoperative morbidity was observed in the group undergoing routine opposed to selective lymphadenectomy (13.63% vs. 8.69%, *p* = 0.36). There was no difference in long-term survival between the groups (HR = 0.90, 95% CI 0.52–1.58, *p* = 0.70). There were also no significant differences in the subgroup of patients with pN0 stage (HR = 1.17, 95% CI 0.6–2.11, *p* = 0.60).

**Conclusions:**

These data suggest that there is no survival benefit from the use of routine lymphadenectomy during surgery for colorectal liver metastasis, but these data should be confirmed in a prospective randomized study.

## Background

The liver is the most common site of metastatic spread after curative treatment of colorectal cancer (CRC), and approximately 50% of all patients with CRC will develop liver metastases [1]. Liver resection represents the best treatment option, with a long-term survival rate of 30%. Resectability is recommended only in approximately 20% of patients, mainly due to the presence of extrahepatic disease [2]. The infiltration of lymph nodes (LN) in the hepatic pedicle and the coeliac region is considered an extrahepatic disease, and many authors still consider its involvement to be a contraindication for liver resection, especially if they are in the coeliac region. Limited data are available on the importance of routine lymphadenectomy of the hepatoduodenal ligament in the literature. Moreover, its survival benefit for both prophylactic and therapeutic indications is not clear [2]. To date, there have been no prospective randomized studies published on this topic. The combination of two different surgical teams with different approaches to lymphadenectomy during liver resection in CRC, at the National Cancer Institute Bratislava Slovakia (NCI), gave us the opportunity to conduct a study with the aim of evaluating the role of routine lymphadenectomy during liver resection in patients with CRC.

## Methods

This retrospective cohort study was conducted to determine the influence of routine lymphadenectomy of the hepatoduodenal ligament on postoperative morbidity and long-term survival in patients with clinically negative nodal status in the hepatoduodenal ligament according to preoperative computed tomography (CT) scans.

### Patient selection

**Fig. 1:**
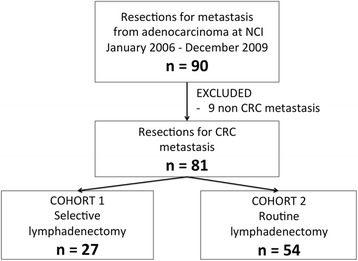
Patients’ selection

From January 2006 to December 2009, eighty-one patients underwent liver resection due to CRC metastases at the National Cancer Institute Bratislava. Patients were divided into two cohorts: patients with or without a routine lymphadenectomy (Fig. 1). This was possible due to the existence of two different hepatobiliary surgical teams working during that time period in the NCI with different approaches to lymphadenectomy. At that time, the presence of nodal involvement in the hepatic pedicle and coeliac region was considered a contraindication for hepatic resection, so all patients who underwent liver resection had clinically negative nodes. At that time, no patient received neoadjuvant or “conversion” chemotherapy before hepatic resection; thus, its influence is of no concern for survival analysis. Administration of adjuvant chemotherapy was considered individually, on the basis of an overall assessment of the below-mentioned prognostic factors. The most commonly used regimen was fluoropyrimidine plus oxaliplatin. Unfortunately different institutions in Slovakia delivered subsequent oncological treatment, so we did not have data about adjuvant chemotherapy available in all patients.

### Data collection

From the patient’s medical records and the National Center of Medical Information and Population Register, we constructed specific case report forms with clinical features and survival information. These features included age, gender, American Society of Anesthesiologists (ASA) score, number and size of liver metastases, disease-free interval (from the treatment of the CRC primary tumor), level of preoperative carcinoembryonic antigen (CEA), positivity of pathological nodal staging (pN+) of the primary tumor, definitive histology finding (only hematoxylin and eosin staining was used for the evaluation of nodal status), presence of postoperative complications and their classification according to the Clavien Dindo system [3], 30-day postoperative mortality and overall survival.

### Surgical technic

In cohort 1, no standard lymphadenectomy was performed; nodal sampling (selective lymphadenectomy) was performed only if there was a suspicion for nodal involvement according to macroscopic assessment or intraoperative palpation. In cohort 2, routine lymphadenectomy that included nodes in hepatoduodenal ligament and along the common hepatic artery, coeliac trunk and retropancreatic region was performed.

### Statistical analysis

Statistical relationships were evaluated by different tests depending on the data characteristics (Kolmogorov-Smirnov, *t* test, Fisher-Freeman-Halton test), and Kaplan-Meier’s method and Log-rank test were used to evaluate differences between the groups in terms of survival analysis. All of the statistical hypotheses were tested with a significance level of alpha = 0.05, and the data cut-off for survival analysis was 31 May 2016. The study was approved by the institutional review board of the NCI Bratislava, Slovakia, and a waiver of consent was granted.

## Results

### Cohort characteristics

There were 55 men (66.7%) and 26 women (33.3%) included in the study. The median age was similar in both cohorts: 57 (42–88) versus 61 (38–82) years old, respectively (*p* = 0.63). The ASA score was also not different between the groups (*p* = 0.42). The most important and well-accepted prognostic criteria (metastasis size, number of metastases, preoperative CEA level, disease free interval from the treatment of the primary CRC and the nodal status of primary CRC), as counted both separately and by Fong’s clinical score [4], were equal and exhibited no significant differences (*p* = 0.08). All of the data are shown in Table 1. The cohorts were well balanced regarding known prognostic factors. The mean number of lymph nodes harvested in patients with routine lymphadenectomy was 4.6 (2–13) per patient. Overall, only one lymph node was sampled in 5 patients in the cohort with selective lymphadenectomy.

**Table 1:** Cohort comparisons with no significant differences in any prognostic factors

	Cohort 1	Cohort 2	p value
	Selective lymphadenectomy	Routine lymphadenectomy	
	*n* = 27	*n* = 54	
Age median (min/max)	57 (42–88)	61 (38–82)	0.63^a^
ASA score (1/2/3/4) %	5/68/27/0	18/68/14/0	0.42^a^
Fong’s score (0–2)/(3–5) %	96.97/3.03	83.33/16.67	0.08^b^
MTS size (mm) median (min/max)	21 (10–40)	20 (8–130)	0.12^a^
MTS number median (min/max)	1 (1–8)	2 (1–7)	0.25^a^
CEA level μg/l median (min/max)	5.7 (0.3–850)	6.62 (0.4–850)	0.97^a^
pN+ primary %	41.67	29.09	0.29^a^
disease free < 12 month %	70.37	74.07	0.14^a^

^a^t test

^b^Fisher-Freeman-Halton test

### Postoperative morbidity

We found a higher rate of postoperative complications in the group of patients undergoing routine lymphadenectomy (13.63%) compared to the group of patients with selective lymphadenectomy (8.69%), but this difference did not reach statistical significance (*p* = 0.36).

The severity of complications evaluated by Clavien Dindo classification system also showed no significant differences (*p* = 0.61).

### Survival

The median length of follow up was 61.67 (0.33–122.93) months.

**Fig. 2:**
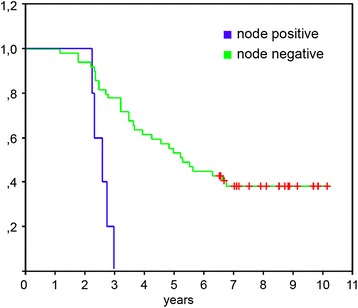
Survival according to histological nodal status. Kaplan-Meier curves showing the difference in overall survival in patients with positive lymph nodes in hepatic pedicle (*n* = 7) compared to patients with negative nodes (*n* = 74). The *green line* indicates patients with negative nodes, the *blue line* patients with positive nodes. The vertical lines on the curve mark censored data. The difference is statistically significant HR 6.33 95% CI 2.16–18.57, *p* = 0.0001

There were 6 patients with positive nodes in cohort 2 (routine lymphadenectomy) and 1 patient with positive nodes in cohort 1 (selective lymphadenectomy), for a prevalence of 11.11% and 3.7% of histological nodal positivity, respectively. Using the Kaplan-Meier method and Log-rank test, we found a significant difference in survival between the patients with positive and negative nodes (median overall survival (mOS) 29.6 vs. 66.97 months, respectively, hazard ratio (HR) 6.33 95% CI 2.16–18.57, *p* = 0.0001) (Fig. 2).

**Fig. 3:**
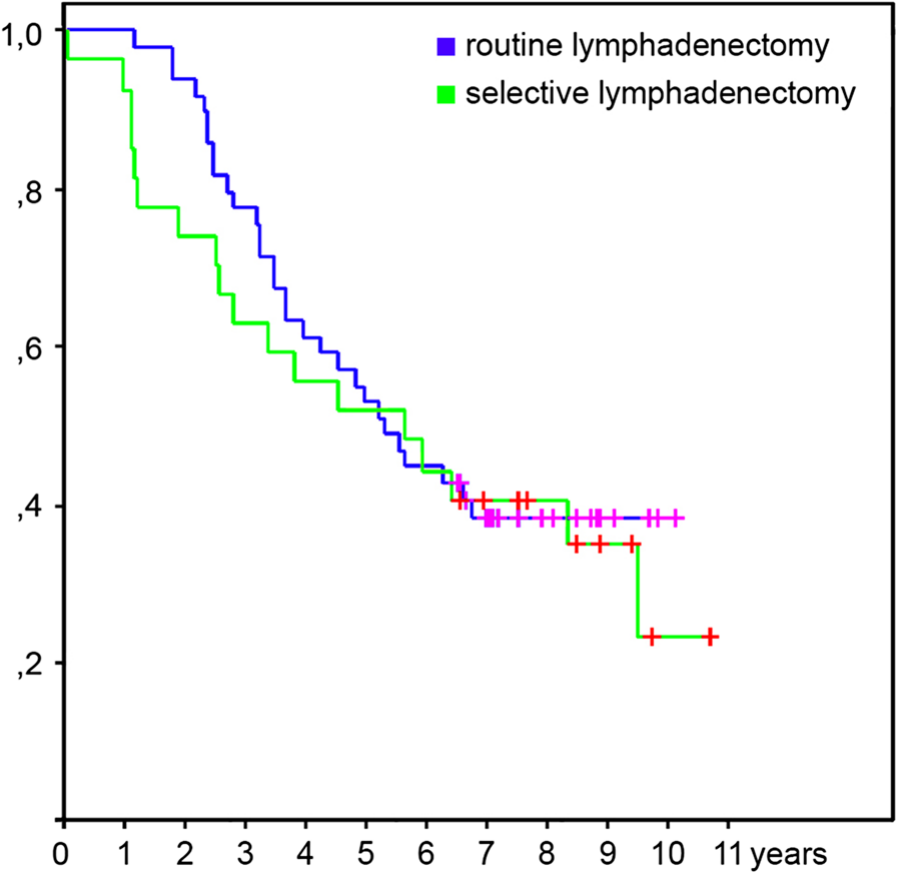
Survival for selective vs. routine lymphadenectomy in all patients. Kaplan-Meier curves showing the difference in overall survival between patients’ cohorts with selective (*n* = 27) and routine (*n* = 54) lymphadenectomy. The *green line* indicates cohort with selective and the *blue line* indicates cohort with routine lymphadenectomy. The vertical lines on the curve mark censored data. Using Log-rank test we found no statistical difference between cohorts HR 0.90 95% CI 0.52–1.58, *p* = 0.7

There were no significant differences in long-term survival between cohorts 1 and 2 (median survival 61.62 vs. 60.16 months, respectively, HR 0.90 95% CI 0.52–1.58, *p* = 0.7) (Fig. 3). The 3- and 5-year survival rates were 59.25 and 51.85% vs. 66.67 and 48.15% in cohorts 1 and 2, respectively.

**Fig. 4:**
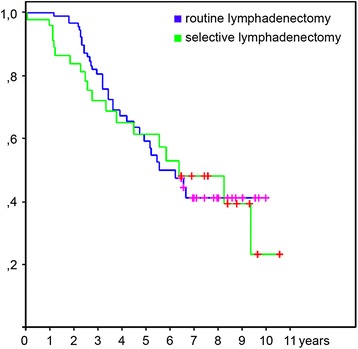
Survival for selective vs. routine lymphadenectomy in pN0 patients. Kaplan-Meier curves showing the difference in overall survival in the subgroup of patients with confirmed pN0 stage. The *green line* indicates cohort with selective and the *blue line* indicates cohort with routine lymphadenectomy. The vertical lines on the curve mark censored data. Using Log-rank test we found no significant difference in survival HR 1.17 95% CI 0.6–2.11, *p* = 0.6 between patients’ cohorts with selective (*n* = 26) and routine (*n* = 48) lymphadenectomy

To evaluate the prophylactic potential of lymphadenectomy, we selected patients with pN0 nodal status after definitive histology and found no significant difference in survival (HR 1.17 95% CI 0.6–2.11, *p* = 0.6) (Fig. 4).

## Discussion

The indication for lymphadenectomy of the hepatoduodenal ligament during liver resection for CRC metastasis remains controversial. It is still unclear whether the lymph node dissection imparts a survival advantage or merely allows a more complete staging of the disease [2]. The metastatic involvement of nodes is considered one of the worst prognostic factors [5, 6] and was once a contraindication for liver resection because the 5 year survival ranged between 0 and 4.3% [7, 8]. The location of the involved nodes also played an important role, as stated by Adam et al: for those who had metastases in the hepatic pedicle and underwent hepatectomy, the 5 year survival was better (25%) than that observed in patients with positive nodes in the coeliac and paraaortic regions (0%) [9]. In our study, we performed en bloc lymphadenectomy, so the subclassification of nodes to different stations was not possible, but none of our node-positive patients survived for 3 years, despite the use of adjuvant chemotherapy. We can therefore confirm that nodal positivity is a negative prognostic factor: node-positive patients exhibited a median survival of 29.6 months vs. 66.97 for node-negative patients.

The overall prevalence of positive lymph nodes was reported to be 8.4%, ranging between 5.4 and 27.9% after routine lymphadenectomy and between 4.0 and 22.5% after selective lymphadenectomy. Koti et al. reported that the prevalence was not correlated to the extent or approach (routine/selective) of lymphadenectomy [10]. In our study, the prevalence was higher in the group of patients undergoing routine lymphadenectomy compared to selective lymphadenectomy (11.11% vs. 3.7%), which can be explained by patient selection for those with cN0 stage. During the study period, the clinical positivity of extrahepatic nodes was considered a contraindication for liver resection.

Regardless of the extent of lymphadenectomy that was performed, we found equal survival in patients with and without routine lymphadenectomy (median survival 61.62 vs. 60.16 months, respectively, HR 0.90 95% CI 0.52–1.58, *p* = 0.7).

To evaluate the “prophylactic” potential of lymphadenectomy, we excluded all patients with proven positive nodes. We assumed, that routine lymphadenectomy in patients with pN0 nodal status could improve survival through the eliminating potential site of future recurrence, as in the cohort with selective lymphadenectomy, patients were expected to have residual nodal disease. We also found no survival advantage of routine lymphadenectomy over no lymphadenectomy (HR 1.17 95% CI 0.6–2.11).

Although the routine lymphadenectomy of the hepatoduodenal ligament in the treatment of liver metastasis from CRC is, according to our results, a safe procedure with acceptable morbidity that is not higher than after liver resection only (*p* = 0.36), we found no survival benefit from its use. Therefore, we suggest considering routine lymphadenectomy during liver resection individually until evidence from high-quality studies supports its use.

Our results do not support the use of routine lymphadenectomy; therefore, we suggest to not perform it as a standard procedure, although its morbidity is the same as after liver resection only (*p* = 0.36). The quality of data in the literature is unclear, and the conclusions are controversial; thus, we suggest that patients be enrolled in prospective clinical studies, which we are currently performing.

## Conclusion

Although the routine lymphadenectomy of the hepatoduodenal ligament in the treatment of liver metastasis from CRC is, according to our results, a safe procedure with acceptable morbidity, we found no survival benefit from its use. Therefore, we suggest considering routine lymphadenectomy during liver resection individually until evidence from high-quality studies supports its use. We confirmed the presence of nodal metastasis as a negative prognostic marker with zero percent 3-year survival.

## Abbreviations

ASA: American Society of Anesthesiologists
CEA: Carcinoembryonic antigen
CRC: Colorectal cancer
CT: Computed tomography
HR: Hazard ratio
LN: Lymph nodes
NCI: National Cancer Institute Bratislava Slovakia
pN+: positivity of pathological nodal staging
vs.: versus
